# Genome sequence of *Desulfobacterium autotrophicum* HRM2, a marine sulfate reducer oxidizing organic carbon completely to carbon dioxide

**DOI:** 10.1111/j.1462-2920.2008.01825.x

**Published:** 2009-05

**Authors:** Axel W Strittmatter, Heiko Liesegang, Ralf Rabus, Iwona Decker, Judith Amann, Sönke Andres, Anke Henne, Wolfgang Florian Fricke, Rosa Martinez-Arias, Daniela Bartels, Alexander Goesmann, Lutz Krause, Alfred Pühler, Hans-Peter Klenk, Michael Richter, Margarete Schüler, Frank Oliver Glöckner, Anke Meyerdierks, Gerhard Gottschalk, Rudolf Amann

**Affiliations:** 1 Göttingen Genomics Laboratory, Georg-August-UniversityGrisebachstr. 8, D-37077 Göttingen, Germany; 2 Max Planck Institute for Marine MicrobiologyCelsiusstr. 1, D-28359 Bremen, Germany; 3 Institute for Chemistry and Biology of the Marine Environment (ICBM), Carl von Ossietzky University OldenburgCarl-von-Ossietzky Str. 9-11, D-26111 Oldenburg, Germany; 4 Center for Biotechnology (CeBiTec), Bielefeld UniversityUniversitätsstr. 37, D-33615 Bielefeld, Germany; 5 Lehrstuhl für Genetik, Fakultät für Biologie, Universität BielefeldD-33594 Bielefeld, Germany; 6 DSMZ – Deutsche Sammlung von Mikroorganismen und Zellkulturen GmbHInhoffenstraße 7 B, D-38124 Braunschweig, Germany

## Abstract

Sulfate-reducing bacteria (SRB) belonging to the metabolically versatile *Desulfobacteriaceae* are abundant in marine sediments and contribute to the global carbon cycle by complete oxidation of organic compounds. *Desulfobacterium autotrophicum* HRM2 is the first member of this ecophysiologically important group with a now available genome sequence. With 5.6 megabasepairs (Mbp) the genome of *Db. autotrophicum* HRM2 is about 2 Mbp larger than the sequenced genomes of other sulfate reducers (SRB). A high number of genome plasticity elements (> 100 transposon-related genes), several regions of GC discontinuity and a high number of repetitive elements (132 paralogous genes Mbp^−1^) point to a different genome evolution when comparing with *Desulfovibrio* spp. The metabolic versatility of *Db. autotrophicum* HRM2 is reflected in the presence of genes for the degradation of a variety of organic compounds including long-chain fatty acids and for the Wood–Ljungdahl pathway, which enables the organism to completely oxidize acetyl-CoA to CO_2_ but also to grow chemolithoautotrophically. The presence of more than 250 proteins of the sensory/regulatory protein families should enable *Db. autotrophicum* HRM2 to efficiently adapt to changing environmental conditions. Genes encoding periplasmic or cytoplasmic hydrogenases and formate dehydrogenases have been detected as well as genes for the transmembrane TpII-*c*_3_, Hme and Rnf complexes. Genes for subunits A, B, C and D as well as for the proposed novel subunits L and F of the heterodisulfide reductases are present. This enzyme is involved in energy conservation in methanoarchaea and it is speculated that it exhibits a similar function in the process of dissimilatory sulfate reduction in *Db. autotrophicum* HRM2.

## Introduction

Shelf sediments receive the highest input of organic carbon among marine systems. They are mostly anoxic and more than 50% of the mineralization of the organic carbon is coupled in these environments to bacterial sulfate reduction (Jørgensen, 1982; Canfield *et al*., 1993). Typically, sulfate-reducing bacteria (SRB) utilize small reduced molecules mostly fermentation products, e.g. acetate, lactate or ethanol, placing them together with syntrophs and methanogens at the end of the anaerobic food chain (Widdel, 1988; Rabus *et al*., 2000). The observed high mineralization rates coupled to bacterial sulfate reduction are indicative for complete oxidation of organic substrates to CO_2_ (Fenchel and Jørgensen, 1977; Jørgensen, 1982). While the frequently isolated and intensively studied *Desulfovibrio* spp. oxidize organic substrates only to the level of acetate, complete oxidation was first demonstrated for *Desulfotomaculum acetoxidans* (Widdel and Pfennig, 1977), *Desulfobacter postgatei* (Widdel and Pfennig, 1981) and *Desulfobacterium autotrophicum* HRM2 (Brysch *et al*., 1987). The nutritionally versatile *Db. autotrophicum* HRM2 oxidizes a variety of organic acids and alcohols to CO_2_ and, in addition, is able to grow chemolithoautotrophically with H_2_, CO_2_ and sulfate. While *Desulfobacter* spp. so far investigated employ modified citric acid cycles for terminal oxidation of acetyl-CoA (Brandis-Heep *et al*., 1983), the Wood–Ljungdahl pathway is used by *Db. autotrophicum* HRM2 (Schauder *et al*., 1986) with acetyl-CoA synthase/CO dehydrogenase (ACS/CODH) as the key enzyme complex. This pathway presumably also functions in the reductive direction for CO_2_ fixation under autotrophic growth conditions (Länge *et al*., 1989; Schauder *et al*., 1989). Corroborating their ecophysiological role members of the *Desulfobacteriaceae* were repeatedly observed to dominate the population of SRB in various anoxic habitats, while *Desulfovibrio* species were mostly absent (e.g. Teske *et al*., 1998; Llobet-Brossa *et al*., 2002; Dhillon *et al*., 2003). Recognition of the environmental significance of SRB resulted in several genome-sequencing projects (for overview see Rabus and Strittmatter, 2007). *Desulfobacterium autotrophicum* HRM2 is the first representative of the ecophysiologically important group of the completely oxidizing sulfate reducers. The genome sequence presented here reflects its high metabolic versatility and reveals novel insights into the bioenergetics of dissimilatory sulfate reduction.

## Results and discussion

### General genome features

#### Genome size and coding sequences

The genome of *Db. autotrophicum* HRM2 consists of two circular replicons, a chromosome of 5 589 073 base pairs (bp) encoding 4871 CDS (Accession No. CP001087) and a plasmid (pHRM2a) of 62 962 bp encoding 76 CDS (CP001088). Three of these encode proteins for plasmid maintenance of the Par family, the remaining CDS only share weak similarities with other proteins. Therefore, the plasmid does not carry any known physiological function. With 5.5 megabasepairs (Mbp) the chromosome of *Db. autotrophicum*HRM2 is about 2 Mbp larger than those of the other three δ-proteobacterial SRB with to date published genomes: *Desulfotalea psychrophila* LSv54 (Rabus *et al*., 2004), *Desulfovibrio vulgaris* Hildenborough (Heidelberg *et al*., 2004) and *Desulfovibrio desulfuricans*G20 (Copeland *et al*., 2005). The principal features of the *Db. autotrophicum*HRM2 genome in comparison with other sequenced sulfate- and sulfur-reducing prokaryotes are summarized in Table 1. A general overview is given in the Fig. S1.

**Table 1:** General genome features of *Db. autotrophicum* HRM2, of four other δ-proteobacterial sulfate reducers, of *G. sulfurreducens* and of the archaeon *A. fulgidus.*

Genome feature	*Db. autotrophicum* HRM2		*Dt. psychrophila* LSv54			*Dv. vulgaris* Hildenborough		*Dv. desulfuricans* G20	*G. sulfurreducens* PCA	*A. fulgidus* VC-16	*Dc. oleovorans* Hxd3
Replicon	Chrom.	Plasmid	Chrom.	Large plasmid	Small plasmid	Chrom.	Plasmid	Chrom.	Chrom.	Chrom.	Chrom.
Size (bp)	5 589 073	62 962	3 523 383	121 587	14 664	3 570 858	202 301	3 730 232	3 814 139	2 178 400	3 944 167
G+C content (mol%)	48.9	42.0	46	43	28	63	66	58	60	48	56
Stable RNAs											
rRNAs	19	–	22	–	–	15	–	12	6	3	3
tRNAs	51	–	64	–	–	67	1	66	49	46	46
Coding sequences (CDS)	4 871	76	3 116	101	17	3 379	152	3 775	3 446	2 420	3 265
Coding (%)	89	79	85	77	72	86	85	90	90	91	n.d.
Assigned functions (%)	71	41	54	60	24	63	63	61	68	48	n.d.
Hypothetical proteins (%)	29	59	46	40	76	37	37	39	32	62	n.d.
Paralogues genes (Mbp^−1^)	132	n.d.	35	n.d.	n.d.	39	n.d.	94	77	87	n.d.
Orthologues genes	*ref*	–	1 534	n.d.	n.d.	1 472	n.d.	1 526	1 428	681	1 688
% of *Db. auto* in X	*ref*	–	31	n.d.	n.d.	30	n.d.	31	24	14	34
% of X in *Db. auto*	*ref*	–	47	n.d.	n.d.	42	n.d.	40	34	28	51
Selenocysteine- containing proteins	31	0	9	0	0	8	1	n.d.	14	0	0

n.d., no data; *Db., Desulfobacterium, G*., *Geobacter, Dt*., *Desulfotalea, Dv., Desulfovibrio, A*., *Archaeoglobus, Dc., Desulfococcus*; *ref*, reference genome for BiBaG (A. Wollherr and H. Liesegang, pers. comm.)*.*

#### Paralogous proteins and repeats

The *Db. autotrophicum* HRM2 genome contains 2357 exact DNA repeats greater than or equal to 50 bp, giving an average of 422 repeats per Mbp (Mbp^−1^). The total number of genes with one or more paralogues is 1460. This corresponds to 265 genes with paralogues per Mbp, and is the highest number of repetitive DNA stretches and the highest number of paralogous genes in all compared genomes (Table 1, Tables S1 and S2). The distribution of the number of paralogous genes is strongly biased, e.g. key enzymes of the sulfate reduction pathway, CO_2_ fixation and central metabolism are unique or have only few copies (≤ 3). This is in clear contrast to genes for substrate utilization where for instance 11 paralogues of acyl-CoA synthetases and 17 paralogues of acyl-CoA dehydrogenases could be identified. The presence of multiple copies of metabolic genes could represent an ecological advantage allowing an expansion of functional capabilities in response to varying environmental conditions (e.g. redox states or substrate concentrations).

#### Comparative genomics

A bidirectional blast comparison of *Db. autotrophicum* HRM2 with *Dt. psychrophila* LSv54 (Rabus *et al*., 2004), *Dv. desulfuricans* G20 (Copeland *et al*., 2005), *Dv. vulgaris* Hildenborough (Heidelberg *et al*., 2004), *Archaeoglobus fulgidus* VC-16 (Klenk *et al*., 1997), *Geobacter sulfurreducens* PCA (Methé*et al*., 2003) and *Desulfococcus oleovorans* Hxd3 (Copeland *et al.*, 2008) revealed that these organisms share between 681 and 1688 orthologous proteins (Table 1). However, genome alignments did not show significant synteny between *Db. autotrophicum* HRM2 and any other SRB. Apparently, there is no comprehensive ‘core’-genome characteristic for all sulfate-reducing prokaryotes (see also Fig. S2).

### Carbon metabolism

#### Fatty acids

**Fig. 1:**
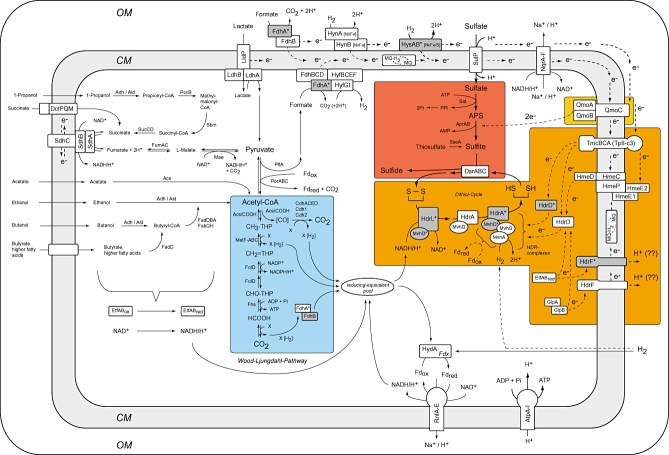
Metabolic reconstruction of *Db. autotrophicum* HRM2 based on known growth substrates and metabolic capacities. Complete oxidation of organic substrates and CO_2_ fixation under autotrophic growth conditions proceeds via the Wood–Ljungdahl pathway (blue box). The sulfate reduction is given in a dark red box, heterodisulfide reduction is given in a dark orange box and electron transfer from Qmo to sulfate reduction is given in an orange box. All selenium-dependent proteins are marked by dark grey backgrounds and an asterisk (*). The cytoplasmic membrane is given in light grey. Arrows indicate metabolic flows; dashed lines indicate assumed or putative electron flows. For reasons of simplicity, cytochromes, multihaem proteins and the following proteins with one or more paralogues are displayed only once in the figure: Acs, CysAW, EtfAB, FdhAB, GlpAB, HdrA, HdrL, MvhD, PorABC, RnfA–E, Sat, Suc, SulP, TmcBCA. Abbreviations are: *CM*, cytoplasmic membrane; *OM*, outer membrane; Acs/CODH, acetyl-CoA synthase/CO dehydrogenase; Acs, acetyl-CoA synthetase; AcsF, CODH maturation factor; Adh, aldehyde dehydrogenase; AMP/ADP/ATP, adenosine-monophosphate/-diphosphate/-triphosphate; AprAB, adenylylsulfate reductase; AtpA–I, ATP synthase F0/F1; Cit, citrate synthase; CO_2_, carbon dioxide; Cdh1/2, carbon monoxide dehydrogenase; CdhA–E, bifunctional acetyl-CoA synthetase/carbon monoxide dehydrogenase; DctPQM, TRAP-type C4-dicarboxylate transporter; DsrABC, dissimilatory sulfite reductase complex; EtfAB_ox_/EtfAB_red_, electron transfer flavoprotein, oxidized and reduced form; FabGH, 3-oxoacyl-acyl carrier synthase/reductase; FadDBA, long-chain fatty acid-CoA ligase; FdhABCD, formate dehydrogenase; Fd_ox_/Fd_red_, ferredoxin, oxidized and reduced form; FdrABC, fumarate reductase; Fhs, formate-tetrahydrofolate ligase; Fdx, ferredoxin, 4Fe-4S cluster; Fhs, formate-tetrahydrofolate ligase; FolD, methylenetetrahydrofolate dehydrogenase; FumAC, fumarate hydratase; GlpAB, glycerol-3-phosphate dehydrogenase; HdrADFL, heterodisulfide reductase; HmeCDEP, Hdr-like menaquinol-oxidizing complex; HydA, [Fe]-only fusion hydrogenase; HyfBCDEFGI, hydrogenase Hyf homologue; HynAB, periplasmic [Ni/Fe] hydrogenase; HysAB, periplasmic [Ni/Fe/Se] hydrogenase; Idh, isocitrate dehydrogenase; LdhAB, lactate dehydrogenase; LldP, l-lactate permease; Mae, malic enzyme; MetF-ABC, methylenetetrahydrofolate reductase; MQ/MQ-H_2_, menaquinone pool, oxidized and reduced form; MvhADG, methylviologen non-reducing hydrogenase; NAD^+^, NADH/H^+^, nicotinamide-adenine dinucleotide, oxidized and reduced form; NADP^+^, NADPH/H^+^, nicotinamide-adenine dinucleotide phosphate, oxidized and reduced form; NqrA–F, Na^+^-translocating NADH-quinone reductase; PccB, propionyl-CoA carboxylase; Pfl, pyruvate formate lyase; PorABC, pyruvate:ferredoxin oxidoreductase; PPi, pyrophosphate; QmoABC, quinone-interacting membrane-bound oxidoreductase complex; RnfA–E, electron transport complex protein; Sat, sulfate adenylyl transferase; Sbm, methylmalonyl-CoA mutase; SdhABC, succinate dehydrogenase/fumarate reductase; SseA, thiosulfate sulfur transferase; SucCD, succinyl-CoA synthetase; SulP, high-affinity H^+^/SO_4_^2−^ symporter; TmcABC, acidic type II cytochrome complex; Tst, thiosulfate sulfur transferase; X/XH_2_, unknown carrier of reducing equivalent, oxidized and reduced form.

**Fig. 2:**
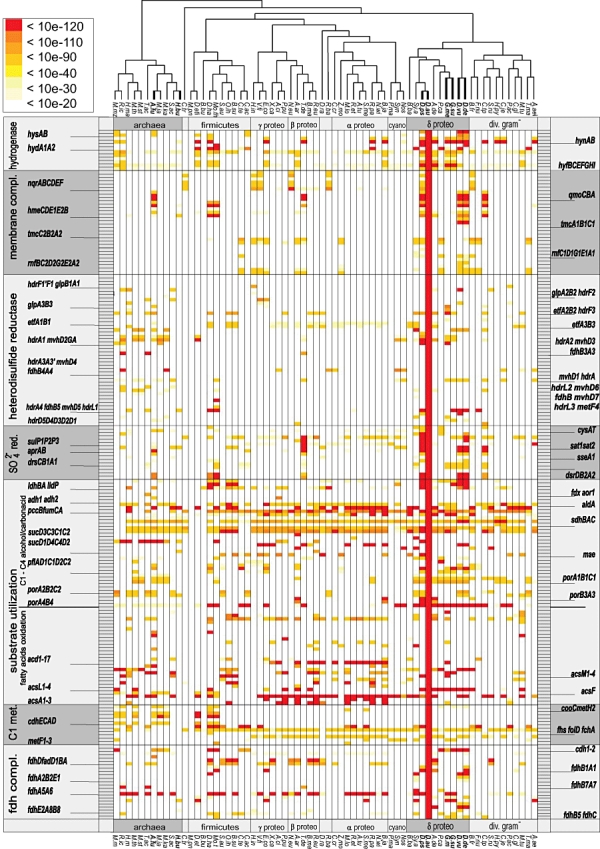
Metabolic heat map of *Db. autotrophicum* HRM2 (white-orange-red scale on similarity; metabolic genes versus organisms). In the red column 206 metabolic genes of *Db. autotrophicum* HRM2 are grouped into seven functional categories: hydrogenase, membrane complexes, *hdr* genes, sulfate reduction, substrate utilization, C1-metabolic pathway and formate dehydrogenases. First and best bidirectional alignments with the proteins of 67 phylogenetically important, but taxonomically diverse prokaryotes are given by colour-coded boxes. Colours correspond to similarity as indicated on the top left panel. A white background indicates no similarities and no hits, dark-red boxes indicate high similarities and best hits. The grey ladders on the left and the right side indicate the 206 genes compared. Paralogous genes in *Db. autotrophicum* HRM2 were not indicated by name but by corresponding boxes, e.g. 17 grey boxes for acd1–17. The complete bidirectional blast data with 700 to date sequenced prokaryotes genomes and the 4947 genes of *Db. autotrophicum* HRM2 are given in Table S1. Order of organism abbreviations follows the grouping used in this figure: Archaea:*M.mz, Methanosarcina mazei*; *R.ic*, Rice cluster RC1; *H.ma, Haloarcula marismortui* ATCC 43049; *M.th, Methanothermobacter thermoautotrophicus* str. ΔH; *M.st, Methanosphaera stadtmanae*; *T.ac, Thermoplasma acidophilum*; *A.fu, Archaeoglobus fulgidus*; *M.ja, Methanococcus jannaschii*; *M.ka, Methanopyrus kandleri*; *S.ac, Sulfolobus acidocaldarius* DSM 639; *H.bu, Hyperthermus butylicus*; Chlamydiae: *C.tr, Chlamydia trachomatis*; Firmicutes: *M.pn, Mycoplasma pneumoniae*; *D.et, Dehalococcoides ethenogenes* 195; *B.bu, Borrelia burgdorferi*; *D.ha, Desulfitobacterium hafniense* Y51; *Mo.th, Moorella thermoacetica* ATCC 39073; *S.au, Staphylococcus aureus* MRSA252; *O.ih, Oceanobacillus iheyensis*; *B.su, Bacillus subtilis* 168; *C.te, Clostridium tetani* E88; *C.ac, Clostridium acetobutylicum*; γ-proteobacteria: *H.in, Haemophilus influenzae*; *V.fi, Vibrio fischeri* ES114; *E.co, Escherichia coli* K12; *X.ca, Xanthomonas campestris*; *A.ci, Acinetobacter* sp. ADP1; *P.pu, Pseudomonas putida* KT2440; β-proteobacteria: *N.eu, Nitrosomonas europaea*; *A.ar*.,*‘Aromatoleum aromaticum’* EbN1; *T.de, Thiobacillus denitrificans* ATCC 25259; *B.ma, Burkholderia mallei* ATCC 23344; *R.eu, Ralstonia eutropha* JMP134; Deinococcus-Thermus: *D.ra, Deinococcus radiodurans*; Planctomycetes: *R.ba, Rhodopirellula baltica*; α-proteobacteria: *C.cr, Caulobacter crescentus*; *Z.mo, Zymomonas mobilis* ZM4; *M.lo, Mesorhizobium loti*; *R.et, Rhizobium etli* CFN 42; *A.tu, Agrobacterium tumefaciens* C58; *S.me, Sinorhizobium meliloti*; *R.pa, Rhodopseudomonas palustris* CGA009; *N.wi, Nitrobacter winogradskyi* Nb-255; *B.ja, Bradyrhizobium japonicum*; Cyanobacteria: *P.ma, Prochlorococcus marinus* CCMP1375; *Syn, Synechocystis* PCC6803; *Nos, Nostoc* sp.; α-proteobacteria: *B.ba, Bdellovibrio bacteriovorus*; *Sy.a, Syntrophus aciditrophicus* SB; *D.ps, Desulfotalea psychrophila* LSv54; *D.au, Desulfobacterium autotrophicum* HRM2; *A.de, Anaeromyxobacter dehalogenans* 2CP-C; *P.ca, Pelobacter carbinolicus*; *G.me, Geobacter metallireducens* GS-15; *G.su, Geobacter sulfurreducens*; *D.vu, Desulfovibrio vulgaris* Hildenborough; *D.de, Desulfovibrio desulfuricans* G20; diverse Gram-negative bacteria: *B.fr, Bacteroides fragilis* NCTC 9434; *F.nu, Fusobacterium nucleatum*; *C.tp, Chlorobium tepidum* TLS; *S.ru, Salinibacter ruber* DSM 13855; *H.py, Helicobacter pylori* J99; *C.je, Campylobacter jejuni*; *P.ac, Propionibacterium acnes* KPA171202; *C.gl, Corynebacterium glutamicum* ATCC 13032; *M.tu, Mycobacterium tuberculosis* H37Rv; *T.ma, Thermotoga maritima*; *A.ae, Aquifex aeolicus*.

Important features of the carbon metabolism of *Db. autotrophicum* HRM2 are depicted in Fig. 1. One of the ecologically important features of *Db. autotrophicum* HRM2 is the utilization of fatty acids up to a carbon chain length of 16. These fatty acids are probably taken up by a H^+^-driven symporter, then activated to the respective CoA esters (FadD) and broken down to acetyl-CoA moieties by classical β-oxidation (Bcd, FabGH, FadBGA). *Db. autotrophicum* HRM2 contains at least 17 acyl-CoA dehydrogenase genes (*acd*) and 11 acetyl-CoA synthetase genes (*acs*) with specificities for short-, medium- and long-chain fatty acids. Three sets of genes, one short, one medium and one long chain specific, have homologues in other SRB despite the finding that complete fatty acid degradation has not been found in these organisms (Fig. 2). The remaining sets of genes have orthologues in anaerobic firmicutes (*Moorella thermoacetica* and *Desulfitobacterium hafniense*) and in proteobacteria known for their ability to utilize a broad spectrum of substrates (*Ralstonia eutropha* JMP134, *Pseudomonas putida* and ‘*Aromatoleum aromaticum*’ EbN1) (Fig. 2).

#### Organic acids

*Db. autotrophicum* HRM2 utilizes formate, lactate as well as succinate, fumarate and malate (Brysch *et al*., 1987) and the genes for all required enzymes were detected. The *fdhAD* genes encode for three periplasmic formate dehydrogenases with TAT-signal peptides. Eight genes for cytoplasmic formate dehydrogenases are present, three of which have orthologues in *Archaea*. Genes for enzymes involved in the utilization of the other acids mentioned correspond to the homologous genes in other SRB (see Fig. 2). This is also true for the single alcohol dehydrogenase gene present.

#### Degradation of propanol and uneven fatty acids

The degradation of all these carbon sources necessitates channelling through the methylmalonyl-CoA pathway to convert intermediate propionyl-CoA via succinyl-CoA to acetyl-CoA. A gene coding for a methylmalonyl-CoA mutase (*sbm*) could be identified on the chromosome. blast analysis showed that Sbm has the highest similarity with the α-subunit of methylmalonyl-CoA mutase from *Leptospira interrogans* (Ren *et al*., 2003)*.* It seems that a gene encoding the small β-subunit of methylmalonyl-CoA mutase, which is known in some bacteria (Birch *et al*., 1993; Bott *et al*., 1997), is absent in *Db. autotrophicum* HRM2*.* Thus the enzyme of *Db. autotrophicum* HRM2 could be a homodimeric enzyme as reported for the methylmalonyl-CoA mutase from *Escherichia coli* (Roy and Leadly, 1992). The analysis of the *sbm* gene product revealed the presence of the highly conserved signature sequence RIANT at position 370–376 and a classical binding motive for the cobalamin cofactor, DxHxxG(41)SxL(26–28)GG (Charles and Aneja, 1999). In close proximity of the *sbm* gene all genes required for the other enzymes of the methylmalonyl-CoA pathway are present on the chromosome of *Db. autotrophicum* HRM2 resulting in an operon-like organization.

#### Complete oxidation of acetyl-CoA

**Fig. 3:**
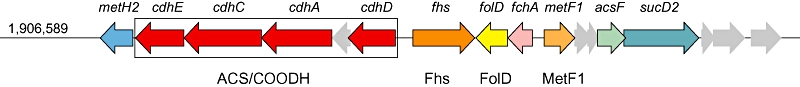
Genomic organization of the Wood–Ljungdahl pathway. In *Db. autotrophicum* HRM2 the genes encoding the key enzymes from the Wood–Ljungdahl pathway are organized in four operon-like structures in a single chromosomal locus. The genes encoding a bifunctional acetyl-CoA synthase/CO dehydrogenase (ACS/CODH) form one colinear group. The genes of the methyl branch of the Wood–Ljungdahl pathway are organized in three distinct groups containing (i) methylene-tetrahydrofolate reductase (MetF1), (ii) methylene-tetrahydrofolate dehydrogenase (FolD) and (iii) C1-tetrahydrofolate synthetase (THF synthetase) (Fhs).

A central element of the metabolic network of *Db. autotrophicum* HRM2 is the ACS/CODH pathway, since complete oxidation of acetyl-CoA to CO_2_ (*heterotrophy*) and CO_2_ fixation (*autotrophy*) proceed via this pathway. A gene cluster could be identified on the chromosome of *Db. autotrophicum* HRM2 encoding all proteins required for the Wood–Ljungdahl pathway to operate in oxidative as well as reductive direction (Fig. 3). The genomic locus is arranged in four operon-like structures. The genes encoding a ACS/CODH form one of these units (classification of CODHs according to Lindahl, 2002). The ACS/CODH protein exhibit the highest similarities to proteins from sulfite-reducing firmicutes, i.e. *Mo. thermoacetica* (Morton *et al*., 1991), *Desulfitobacterium hafniense* (Nonaka *et al*., 2006) and archaea; the complex lacks any orthologues to the SRB from the δ-proteobacterial SRB (Fig. 2). The genes encoding the proteins of the methyl branch of the pathway are organized in three distinct groups containing (i) methylene-tetrahydrofolate reductase (MetF), (ii) methylene-tetrahydrofolate dehydrogenase (FolD) and (iii) C1-tetrahydrofolate synthetase (THF synthetase) (Fhs). Orthologous genes of the methyl branch can be found in all taxonomic groups, but again sulfite-reducing firmicutes and archaea contain the most similar orthologues (Fig. 2).

#### Chemolithoautotrophy

The unique and physiologically important property of *Db. autotrophicum* HRM2 is the ability to grow with H_2_, CO_2_ and sulfate. It already has been mentioned that CO_2_ fixation may proceed via the Wood–Ljungdahl pathway. In addition, two further genes for monofunctional CODHs (*cdh1* and *cdh2*) could be identified at different locations on the chromosome. In contrast to *acs*/*codh*, their genetic context displays no relation to any other proteins of the Wood–Ljungdahl pathway. CODH2 has highly similar orthologues in *Methanosarcina mazei* (Deppenmeier *et al*., 2002), *Mo. thermoacetica* (Morton *et al*., 1991), *Ds. hafniense* (Nonaka *et al*., 2006) and the SRB *Dv. vulgaris* (Heidelberg *et al*., 2004) and *Dv. desulfuricans* (Copeland *et al*., 2005)*.* In *Ms. mazei* the orthologous gene product is the β-subunit of ACS/CODH, which catalyses the reversible oxidation of CO (Deppenmeier *et al*., 2002). Synthesis of pyruvate is then accomplished by one of the eight present pyruvate:ferredoxin oxidoreductases (*por*) and this compound can be further carboxylated to oxaloacetate by the biotin dependent pyruvate carboxylase PcB. The hydrogenases which, of course, are essential for chemolithoautotrophic growth will be discussed in the next session.

### Energy conservation during sulfidogenesis

#### Dissimilatory sulfate reduction

Reduction of sulfate to sulfide can be divided into two steps: (i) the reduction of sulfate to sulfite, which is connected with the conversion of ATP to AMP and pyrophosphate, and (ii) the six-electron reduction of sulfite to sulfide. It was shown by Badziong and Thauer (1978) that bacterial growth yields with sulfite and hydrogen (H_2_) are higher than those with sulfate and hydrogen (H_2_). This is in agreement with an ATP-consuming first step but requires that the second step is coupled to the generation of a protonmotive force, which then can be taken advantage of for ATP synthesis. Sulfate uptake in *Db. autotrophicum* HRM2 is performed via three high-affinity, H^+^-driven symporters (*sulP1–3*), while homologues to the permease subunit CysP of the ABC-type transport system (*cysATP*) are absent. Two genes for ATP sulfurylase (*sat*) as well as genes for the reduction of the adenosine-5′-phosphosulfate (APS) as catalysed by a single APS reductase (*aprAB*) were detected (Fig. 1). Genes encoding the dissimilatory sulfite reductase (*dsrABCD*) converting sulfite to sulfide were shown to be present in two loci on the chromosome. In agreement with the capacity of *Db. autotrophicum* HRM2 to utilize thiosulfate as alternative electron acceptor, the chromosome contains a thiosulfate sulfurtransferase gene (*tst*).

#### Evolutionary aspects of sulfate reduction genes

The key enzymes of dissimilatory sulfate reduction, i.e. ATP sulfurylase (*sat*), APS reductase (*aprAB*) and dissimilatory sulfite reductase (*dsrABC*), are scattered around the chromosome of *Db. autotrophicum* HRM2 (Fig. S1), as has been observed for other sulfate reducers with known genome sequences. However, DNA fragments were recently obtained from uncultured prokaryotes that contained clustered genes of sulfate reduction (Mussmann *et al*., 2005). This finding supports speculations about a metabolic island for the pathway of dissimilatory sulfate reduction (Klein *et al*., 2001; Friedrich, 2002). Moreover, Mussmann and colleagues (2005) speculate that the obtained gene cluster could represent a conserved progenitor that was upon its lateral acquisition increasingly scattered due to genome plasticity of the recipient organism. The latter could be driven by mobile elements, which are highly abundant in the genome of *Db. autotrophicum* HRM2. There are two paralogues of *sat*, whereas the *aprAB* and the *dsrABC* genes are unique in the genome of *Db. autotrophicum* HRM2. This is a surprisingly low number, considering that on average each gene has five paralogues in the genome.

#### Electron donors

**Fig. 4:**
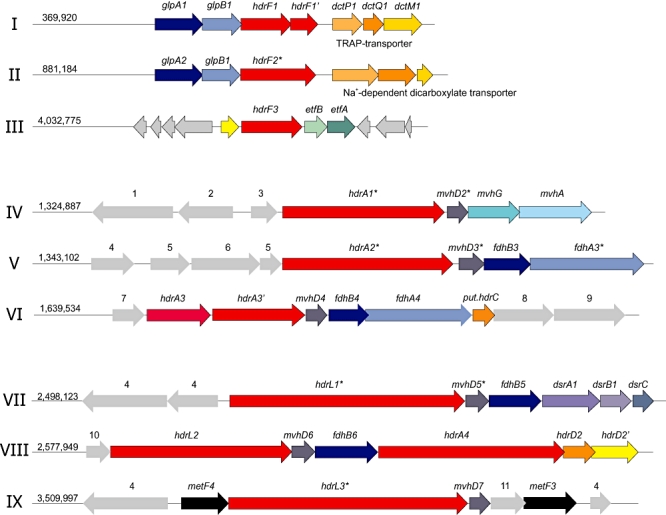
Genomic context of the *hdrA, hdrF, hdrD* and *hdrL* genes of *Db. autotrophicum* HRM2. Deduced heterodisulfide reductase genes are marked in red. The HdrA/HdrL proteins are encoded at nine genetic loci, one of which contains a tandem associated *hdrL*/*hdrA* copy (VIII). Each *hdrA* or *hdrL* locus is associated with a methylviologen non-reducing hydrogenase subunit D (*mvhD*), beside the *hdrA4* locus, which is followed by a deduced *hdrD2* gene activity (VIII). The loci V, VI, VII and VIII contain genes for a formate dehydrogenase subunit B, which is followed directly by formate dehydrogenase subunit A genes in loci V and VI. In locus VIII *hdrA4* gene is associated with genes for deduced orthologues of subunits HdrB and HdrC, giving an *hdrACB*-like operon as can be found in *Desulfovibrio* species. The HdrF1 and HdrF2 genes are associated with genes for anaerobic glycerol-3-phosphate dehydrogenase activity (GlpAB) which probably transfers electrons from dicarboxylic acid degradation and genes for dicarboxylic acids transporters (Dct). *hdrF3* is clustered with electron transfer flavoprotein subunits EtfBA, which might transfer electrons from the β-oxidation of fatty acids. Abbreviations are: *hdrA*, heterodisulfide reductase, subunit HdrA; *hdrL*, predicted heterodisulfide reductase/glutamate synthase fusion protein, subunit HdrL; *hdrF*, heterodisulfide reductase subunit HdrF; *hdrD*, heterodisulfide reductase, subunit HdrD (HdrCB homologous protein); *dsrA*, dissimilatory sulfite reductase, α-subunit; *dsrB*, dissimilatory sulfite reductase, β-subunit; *dsrC*, dissimilatory sulfite reductase, γ-subunit; *mvhD*, methylviologen non-reducing hydrogenase, iron-sulfur subunit MvhD; *mvhG*, methylviologen non-reducing hydrogenase, iron-sulfur subunit MvhG; *mvhA*, methylviologen non-reducing hydrogenase, nickel-iron subunit MvhA; *glpA*, anaerobic glycerol-3-phosphate dehydrogenase large subunit; *glpB*, anaerobic glycerol-3-phosphate dehydrogenase small subunit; *etfA*, electron transfer flavoprotein α-subunit; *etfB*, electron transfer flavoprotein β-subunit; *dctP1* TRAP-type C4-dicarboxylate transporter, periplasmic solute-binding component; *dctM1*, TRAP-type C4-dicarboxylate permease, large subunit; *dctQ1*, TRAP-type C4-dicarboxylate permease, small subunit; 1, HysB, periplasmic [Ni/Fe/Se] hydrogenase, large subunit; 2, HysD, periplasmic [Ni/Fe/Se] hydrogenase, small subunit; 3, HyaD2, hydrogenase expression/formation protein; 4, hypothetical protein; 5, response regulator; 6, histidine kinase; 7, ferredoxin; 8, Fe/S cluster protein; 9, Aor5, tungsten-containing aldehyde reductase; 10, CheY-like regulator; 11, putative regulatory protein.

Genes for one periplasmic [Ni/Fe/Se] hydrogenase (*hysAB*), one periplasmic [Ni/Fe] hydrogenase (*hynAB*), as well as three periplasmic formate dehydrogenases (*fdhAB*) are present in the genome of *Db. autotrophicum* HRM2 (Figs 1 and 2). The [Ni/Fe/Se] hydrogenase is apparently soluble, therefore requiring a soluble *c*-type cytochrome to transfer the electrons to the type II cytochrome *c*_3_ complex for transmembrane passage (Pieulle *et al*., 2005). The putative membrane association of the [Ni/Fe] hydrogenase suggests a direct transfer of electrons derived from hydrogen oxidation onto a thus far unknown transmembrane carrier. All corresponding enzymes are capable of producing scalar protons, and the electrons can be transferred to redox-active complexes such as TpII-*c*_3_ (TmcBCA), Qmo (QmoCAB) or Hme (HmeCDE1E2P) either directly, or indirectly via the menaquinol pool (MQ/MQ-H_2_) (Fig. 1). H_2_ can be produced in the cytoplasm by a formate–hydrogen-lyase complex consisting of FdhABCD and an associated [Ni/Fe] hydrogenase HyfBCEFGI (Hedderich and Forzi, 2005; Pereira *et al*., 2006). Apparently, *Db. autotrophicum* HRM2 does not contain the membrane-bound EchABCDE [Ni/Fe]- or the CO-dependent hydrogenases (CooMLKXUHF), which are enzymatically active towards the cytoplasmic side. Both of these hydrogenases are present in *Dv. vulgaris* Hildenborough (Heidelberg *et al*., 2004) and Ech also in *Desulfovibrio gigas* (Rodrigues *et al*., 2003). The enzymes are assumed to play a prominent role in the proposed hydrogen cycling (Voordouw, 2002; Heidelberg *et al*., 2004). But genes encoding a [Fe]-only hydrogenase (HydA) catalysing ferredoxin reduction with H_2_ and a cytoplasmic selenocysteine-containing hydrogenase (MvhA2DG) are present in *Db. autotrophicum* HRM2. The genes *mvhA2DG* are clustered with the genes for the selenocysteine-containing heterodisulfide reductase HdrA1 and the [Ni/Fe/Se] hydrogenase HysAB (Figs 1 and 4).

**Fig. 5:**
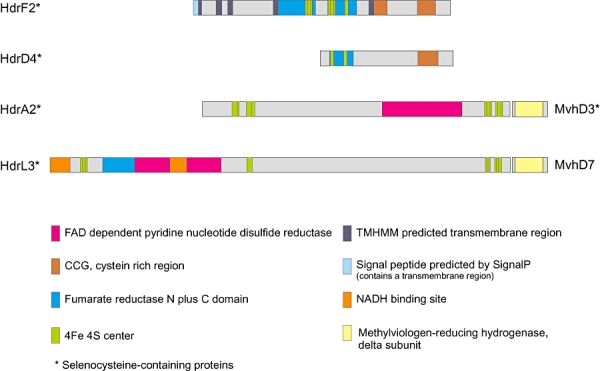
Pfam (domain scans analysis) and domain alignments of four types of heterodisulfide reductase subunits (Hdr) of *Db. autotrophicum* HRM2. Relevant Pfam domains are given in colour codes. All Hdr proteins depicted contain selenocysteine; the HdrA and the HdrL type of the proteins are colocated with methylviologen non-reducing hydrogenase subunit D (mvhD). The deduced HdrF protein contains a domain with several transmembrane regions indicating a possible membrane integration of the protein. In contrast, HdrD, HdrA and HdrL have domain structures typical for soluble proteins.

Electron donors during chemoorganotrophic growth are membrane-bound lactate (LdhAB) and glycerophosphate (GlpAB) dehydrogenases as well as NADH/H^+^ and EtfAB from β-oxidation of organic acids (FadBGA) and various dehydrogenase reactions (Fig. 1). Electrons from the membrane-bound dehydrogenases can be channelled into the quinone pool or to the heterodisulfide reductase subunits (HdrF) either directly or indirectly via the Hme (HmeCDE1E2P) complex. The genomic proximity of two GlpAB gene clusters and an EtfAB gene cluster with the F-type heterodisulfide reductases (HdrF1–HdrF3) supports the idea of an electron transfer via the heterodisulfide reductases. NADH can be oxidized by the NADH-quinone oxidoreductase (NqrA–F), which also will provide electrons for the quinone pool, or possibly by the novel heterodisulfide reductase HdrL1 to HdrL3 (Figs 4 and 5 and Fig. S3A and B).

#### Membrane-bound redox complexes

The electrons, which originate from H_2_, NADH or ETFs, are transferred to redox complexes of which sulfate-reducing prokaryotes are very rich. In recent years, several membrane-bound complexes have been discovered in *Desulfovibrio* and *Archaeoglobus* spp., which could serve this redox function (see Matias *et al*., 2005). The 16-haem cytochrome Hmc of *Dv. vulgaris* (Rossi *et al*., 1993; Czjzek *et al*., 2002), the Hme complex of *A. fulgidus* (Mander *et al*., 2002), the 9Hc complex of *Dv. desulfuricans* (Saraiva *et al*., 2001) and the TpII-*c*_*3*_ complex of *Dv. vulgaris* (Valente *et al*., 2001; Pereira *et al*., 2007) all possess periplasmic cytochrome *c*_*3*_ subunits for the reception of electrons generated by periplasmic enzymes. Genes encoding several cytochrome *c*_*3*_ family proteins and multihaem cytochrome family proteins as well as cytochrome *c* oxidase and cytochrome *c* assembly protein CcmC are present in *Db. autotrophicum* HRM2. This supports the idea that electrons if necessary can be stored within the periplasm as described previously (Heidelberg *et al*., 2004). Genes for an Hmc complex are apparently not present in *Db. autotrophicum* HRM2 but two TpII-*c*_3_ (TmcBCA) and one Hme encoding gene cluster (HmeDCPE1E2) were detected (Figs 1 and 2). In addition, genes for a Qmo complex (QmoCAB) were found, which could accept electrons, transferred from the NADH-quinone oxidoreductase. The Qmo complex of *Dv. desulfuricans* and *Dv. vulgaris* does not contain a periplasmic cytochrome, but was shown to interact with a soluble menaquinone analogue and suggested to function as a menaquinol/APS reductase oxidoreductase (Pires *et al*., 2003; Haveman *et al*., 2004). Similarly, a direct electron transfer from HmeDEAB has been proposed for *Dv. vulgaris* (Haveman *et al*., 2004). Recent experimental studies with *Dv. desulfuricans* ATCC 27774 suggest that Hme channels electrons from the periplasm and menaquinone pool to sulfite reductase (Pires *et al*., 2006). In the obligatory hydrogenotrophic *Archaeoglobus profundus*, a hydrogenase was found to form a tight complex with a soluble heterodisulfide reductase (Mvh:Hdl), supporting the assumed role of heterodisulfide reductase in electron transfer of sulfate-reducing prokaryotes (Mander *et al*., 2004).

#### Role of heterodisulfide reductases (Hdr)

The archaeal Hdr plays a key role during methanogenesis. The enzyme reduces the heterodisulfide of coenzymes M and B which is formed in the methyl-coenzyme M reductase reaction by which methane is produced. Hdr of *Ms. mazei* has been shown to be a membrane protein consisting of the subunits D and E. Receiving the electrons from methanophenazine via cytochromes it functions as a proton pump (Deppenmeier *et al*., 2002). Methanogens growing with H_2_ and CO_2_ but not with acetate or methanol do not contain cytochromes and contain a different Hdr consisting of the subunits ABC, which is also true for *Methanosphaera stadtmanae* growing with methanol and H_2_ (Fricke *et al*., 2006). *In silico* analysis indicates that *Db. autotrophicum* HRM2 may express 15 paralogues of Hdr that can be divided into four protein types and that could form differently composed protein complexes (Figs 1 and 4). As shown in Fig. 4 the nine loci at which Hdr genes occur are spread over the chromosome of *Db. autotrophicum* HRM2. Additionally, Hdrs differ considerably not only in size but also in domain compositions (Fig. 5 and Fig. S3A and B). Four of the Hdr were designated as *hdrA* and share high similarities with the corresponding subunits of the archaea. In addition to the four HdrA subunits of which only HdrA3/A3′ contains a small predicted transmembrane region, a large HdrL type has been defined. Especially the selenocysteine protein HdrL3 is of interest because it contains predicted regions for a fumarate reductase domain, and additionally a NADH binding site, which is also found in the two other HdrL subunits. HdrL therefore may be essential for electron cycling during sulfidogenesis (Fig. 1). All the CDS for HdrAs and HdrLs except HdrA4 are followed by a CDS for the D-subunit of the methylviologen non-reducing hydrogenase (Mvh) (Figs 4 and 5 and Fig. S3A and B). In *Methanothermobacter marburgensis* MvhD and HdrA form a tight complex in which MvhD transfers reducing equivalents to Hdr (Stojanowic *et al*., 2003). Genomic comparisons show that MvhD homologues are colocated with *hdr* genes even in organisms where no complete Mvh hydrogenase is present. In some of these organisms, including the *Methanosarcina* species and *A. fulgidus*, MvhD and HdrA have been fused to a single protein (Fig. S3A). Genes of dissimilatory sulfite reductase subunits DsrA, DsrB and DsrC follow HdrL1 and MvhD (Fig. 4). Thus, the HdrL1 gene cluster encodes all proteins necessary to transfer electrons from NADH/H^+^ to sulfite (SO_3_^2−^).

The *hdrD* genes as present in *Ms. mazei* and *A. fulgidus* (Hedderich *et al*., 2005) could be detected also in *Db. autotrophicum* HRM2. Two variant genetic versions are present within the chromosome. The *hdrD1* to *hdrD4* gene products contain HdrC and HdrB homologue domains, but are lacking transmembrane parts (Fig. 5). Three *hdrD*-like gene products contain both, multiple transmembrane domains as well as domains homologous to HdrC and HdrB. These genes were designated *hdrF1* to *hdrF3*. The protein deduced from the *hdrF2* gene is a selenoprotein; it contains the transmembrane region, the CCG region and the fumarate-reductase domain (Fig. 5). All three *hdrF* genes are clustered with genes encoding electron providing proteins, *hdrF1* and *hdrF2F2′* with *glpAB* and dicarboxylic acid transporters and *hdrF3* with *etfAB* involved in β-oxidation (Fig. 4).

A direct orthologue to *hdrE* of *Ms. mazei* and *Dt. psychrophila* LSv54 is lacking in *Db. autotrophicum* HRM2. Instead, the three present *hdrF* genes (Fig. S3B) are much larger; all genes carry sequence regions for a signal peptide and up to six transmembrane helices. Taking into account the genomic context of the *hdrF* genes and the fact that the Hdrs of some methanoarchaea are deduced proton pumps, one could speculate that this function may also apply to the *hdrF* genes of *Db. autotrophicum* HRM2 (Fig. 1).

#### A concept for electron transfer and energy conservation

When *Db. autotrophicum* HRM2 is growing with H_2_, CO_2_ and sulfate, eight electrons are required for sulfide production. Their generation by periplasmic hydrogenases would result in a maximum of eight scalar protons. The corresponding protonmotive force could give rise to the synthesis of 2^2^/_3_ ATP. However, an equivalent of two ATP is required for the reduction of sulfate to sulfite. In addition, transport of sulfate and other compounds are ATP- or ionmotive force-consuming. The conclusion is that there must be pumps present to generate additional proton or sodium ion gradients. One candidate for such a pump is the RnfA–E cluster. Genes for a hydrogenase catalysing the reduction of ferredoxin were detected. Electron transfer from reduced ferredoxin to NAD^+^ via Rnf would then allow the generation of a proton (or sodium ion) motive force. Proton pumping Rnf clusters have been integrated in concepts of *Clostridium tetani* (Brüggemann *et al*., 2003) and *Clostridium kluyveri* (Seedorf *et al*., 2008) energy metabolism. Further candidates are the various heterodisulfide reductases. They could receive electrons from membrane-bound redox complexes, in case of HdrL from NADH or other electron carriers, and provide the electrons for sulfite reduction (Fig. 1). In analogy to methanogenic archaea it is tempting to speculate that these processes are also coupled to generation of a protonmotive force. Under low partial pressure of H_2_, HydA will not be able to reduce ferredoxin (*E*′ of about −500 mV) and to provide Fd_red_ for the reduction of CO_2_ to CO, for pyruvate synthesis from acetyl-CoA and CO_2_ and for the RnfA–E complex. Under these conditions, the recently discovered electron bifurcation reaction (Thauer *et al*., 2008) may provide the reduced ferredoxin (Fd_red_) required (Fig. 1). This reaction couples the reduction of crotonyl-CoA to butuyryl-CoA with Fd_red_ generation and is the key reaction in clostridia, notably in *C. kluyveri* (Seedorf *et al*., 2008). It also plays a role in energy metabolism of methanogens not containing cytochromes (Thauer *et al*., 2008). In *Db. autotrophicum* HRM2 the MvhADG–HdrA complex (Fig. 1) could also bifurcate electrons coming from H_2_ under low partial pressure to reduce the disulfide and ferredoxin. A possible reaction equation could be:


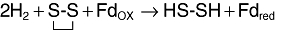


Studies on the proteome of *Db. autotrophicum* and *in vitro* investigations will be required to elucidate the role of the various Hdrs for which the genes have been detected in this work.

### Metabolic heat map

A phylogenetic comparison was performed based on bidirectional blast comparisons of *Db. autotrophicum* HRM2 genes of carbon and energy metabolism with 700 to date available genome data sets (the complete data of the comparisons are given in Table S1). Among the compared were the genomes of 52 archaea including *A. fulgidus* VC-16, of spore formers, chemolithotrophs, phototrophs, enterobacteria and of seven sulfate reducers. Out of the 700, 67 phylogenetically diverse genomes were selected to produce a metabolic heat map (Fig. 2). None of the prokaryotes compared possesses a comparable wealth of genes encoding hydrogenases, heterodisulfide reductases, formate dehydrogenases and enzymes for fatty acid oxidation as *Db. autotrophicum* HRM2. Most of these genes have orthologues in other proteobacteria and firmicutes, but not in the closely related SRB of the δ-proteobacteria. This finding is especially apparent for the heterodisulfide reductases, located at nine genetic loci and present in 15 copies (Fig. 4). The closest phylogenetic relations of the four different Hdrs point to δ-proteobacteria for the HdrF type, to firmicutes for the HdrD type and to sulfate reducers in case of the HdrA genes. The HdrL type has only weak sequence similarities and seems to be unique for *Db. autotrophicum* HRM2. Even the most recently submitted genome of a *Desulfobacteraceae* member, *Dc. oleovorans* Hxd3, (Copeland *et al*., 2008) does not contain an HdrL-type heterodisulfide reductase.

Among the membrane bound redox-active complexes are two *rnf* and one closely related *nqr* system. None of these systems is found in any other SRB (Fig. 2) except *Dc. oleovorans* Hxd3 (Copeland *et al*., 2008). For each of the hydrogenases of *Db. autotrophicum* HRM2 we found orthologous genes in other SRB genomes, but not a single genome contains orthologous genes for all the hydrogenases of *Db. autotrophicum* HRM2. In contrast, the genes for the reduction of sulfite to H_2_S (*aprAB* and *dsrABC*) do not have paralogues in *Db. autotrophicum* HRM2 and have orthologous genes exclusively in other SRB.

### Selenocysteine-containing proteins

Selenocysteine-containing proteins substantially increase the activity of redox-active enzymes as compared with cysteine-containing paralogues. Therefore, selenocysteine-containing proteins are found in many energy-limited anaerobic organisms (Andreesen *et al*., 1999; Gröbe *et al*., 2007). The genome of *Db. autotrophicum* HRM2 encodes the complete selenocysteine translation machinery consisting of *selA, selB, selC* and *selD* and the tRNA^Sec^. Genes for at least 31 selenocysteine-containing proteins were detected, which is more than has been reported for any other SRB genome sequenced (Table 1). Seventeen selenocysteine-containing proteins play a role in energy metabolism of the organism. They encode two heterodisulfide reductases (*hdrA1, hdrA2*), two heterodisulfide reductase fusion proteins (*hdrL1, hdrL3*) (see Fig. 5), one heterodisulfide reductase subunit (*hdrD4*), four cytoplasmic formate dehydrogenases (*fdhA1, fdhA3, fdhA5, fdhA6*), three periplasmic formate dehydrogenases (*fdhA2, fdhA7, fdhA8*), one periplasmic [Ni/Fe/Se] hydrogenase, four F420 non-reducing D-subunit hydrogenases (*mvhD1, mvhD2, mvhD3, mvhD5*) and others (Table S3). The extensive use of selenocysteine-containing proteins is one of the most obvious differences between *Db. autotrophicum* HRM2 and the other SRB with completely sequenced genomes. This may indicate that the *Db. autotrophicum* HRM2 genome encodes proteins that could serve as a functional replacement under selenium limitation as it has been described for the Mvh hydrogenase homologues Vhu (selenocysteine-containing) and Vhc (non-selenocysteine-containing) in *Methanoccous voltae* (Pfeiffer *et al*., 1998).

### Relation to oxygen

While sulfate reducers have long been considered as strict anaerobes, they encounter dynamic changes of oxic/anoxic interfaces in their natural habitat (Jørgensen, 1987). Particularly in the photic zones of microbial mats oxygen-saturated conditions can occur during daytime (Jonkers *et al*., 2005). Early studies indicated that particularly *Desulfovibrio* spp. survive prolonged exposure to oxygen (Cypionka *et al*., 1985). More recent studies also indicated that SRB could in fact respire oxygen as a protective measure (Cypionka, 2000) and migrate into areas of preferential oxygen concentration (Fischer and Cypionka, 2006). The presence of *cydAB* genes coding for a cytochrome *d* ubiquinol oxidase could also enable *Db. autotrophicum* HRM2 to respire oxygen at low concentrations (Green *et al*., 1988; Lemos *et al*., 2001). Additionally, *Db. autotrophicum* HRM2 possesses catalase (Cat), a selenocysteine-containing peroxidase, rubrerythrin (Rbr), and rubredoxin oxidoreductases (Rbo) for oxygen detoxification. The latter two have recently been described as oxygen protection systems in *Dv. vulgaris* (Lumppio *et al*., 2001). However, it should be noted, that subsequent studies revealed no obvious oxidative stress phenotype for an *rbr* mutant (Fournier *et al*., 2003) and downregulation of rubrerythrins in oxygen-exposed cultures of *Dv. vulgaris* (Fournier *et al*., 2006). *Db. autotrophicum* HRM2 possesses several copies of F390 synthetase genes, which has previously been suggested in *Methanothermobacter thermoautotrophicus*ΔH to react to oxygen tension or H_2_ limitation (Morgan *et al*., 1997; Vermeij *et al*., 1997).

### Transcriptional regulation and signal transduction

When looking at its nutritional versatility, *Db. autotrophicum* HRM2 should possess a broad spectrum of adaption capacities, which implicate variable regulations. A search for characteristic protein domains among the group of sensory proteins in the *Db. autotrophicum* HRM2 genome yielded 253 proteins with putative transmembrane sensors (Table S4). Among these are 109 histidine kinases containing the characteristic Pfam domain HATPaseC (Perego and Hoch, 1996). Fifty histidine kinases contain one or more copies of the redox-sensing PAS domain (Zhulin *et al*., 1997; Taylor and Zhulin, 1999), reflecting also the strict dependence of *Db. autotrophicum* HRM2 to react to changes in the oxygen pressure.

ECF-σ-factors (σ^ECF^) have been identified by the presence of characteristic DNA-binding domains (Sigma70_rc2, Sigma70_rc4 and Sigma70_rc4.2) and the absence of a σ^70^-specific domain (Sigma70_rc3). We have identified five alternative σ-factor proteins in *Db. autotrophicum* HRM2, including σ^ECF^ proteins of the RpoE and the SigM type. Additionally, six anti-σ-factors and at least two anti-anti-σ-factors could be identified.

Beside the two-component system respectively ECF-σ-factor-associated proteins 120 proteins were identified which contain sensory protein domains and therefore might represent components of additionally sensory systems.

## Conclusions

Several genome features like larger genome size, higher number of repeated sequence elements and paralogous proteins, extensive use of selenocysteine-containing proteins, and partially closer relatedness to archaea and firmicutes than to other SRB, seem to underline the distinctiveness of *Db. autotrophicum* HRM2 among the SRB with completely sequenced genomes. The genome of *Db. autotrophicum* HRM2 appears to be shaped by many genetic rearrangements, gene duplications and genetic adaptation events, as well as some horizontal gene transfer (HGT). These dynamic processes might have provided the organism with a significantly broader spectrum of orthologous genes for substrate utilization and energy conversion. Oxidizing detritus and the employment of efficient electron transfer systems based on selenocysteine-containing proteins enable this slow-growing species to out-perform the fast-growing and nutritionally limited species of the genus *Desulfovibrio* in the natural habitat.

## Experimental procedures

### Organism

*Desulfobacterium autotrophicum* HRM2 (DSMZ 3382) was obtained from the German Collection of Microorganisms and Cell Cultures (Deutsche Sammlung von Mikroorganismen und Zellkulturen, DSMZ), Braunschweig, Germany.

### Media and cultivation

*Desulfobacterium autotrophicum* HRM2 was cultivated as originally described by Brysch and colleagues (1987), with mineral media corresponding to saltwater medium (Widdel and Bak, 1992). Mineral media were sulfide-reduced (1 mM) and bicarbonate-buffered. Cultivation was carried out at 28°C. For maintenance, cells were grown with 10 mM sodium lactate as sole source of organic carbon and energy and sulfate as electron acceptor. Methods for anaerobic cultivation were performed as described by Widdel and Bak (1992).

### Genome sequencing, assembly and gap closure

Isolation of genomic DNA from *Db. autotrophicum* HRM2 was performed with the Bio-Rad Aqua Pure DNA isolation kit (Bio-Rad, München, Germany). The genomic DNA sequence was determined using three conventional types of whole-genome shotgun libraries of three different insert sizes, as well as two large insert libraries derived from cosmid and fosmid cloning as previously described (Hradecna *et al*., 1998; Wild *et al*., 2002; Schwartz *et al*., 2003). For the whole-genome shotgun libraries DNA fragments of 1.5–2.5, 1.5–3.5 and 2.5–4.0 kb, respectively, were separated by gel electrophoresis after mechanical shearing (Nebulizer; Invitrogen, Carlsbad, USA), end-repaired and cloned using vectors pTZ19R (cmR) (Amersham, Essex, UK), pWE15R (kanR), or pCR2.1-TOPO (TOPO TA Cloning Kit for Sequencing; Invitrogen). PCR fragments were cloned using vector pCRXL-TOPO or pCR4-TOPO (TOPO XL PCR Cloning Kit; Invitrogen). For construction of the cosmid and fosmid libraries, DNA fragments of about 40 kb were isolated using a pulse field electrophoresis system (Chef Mapper; Bio-Rad), and cloned into the SuperCos cosmid vector (SuperCos I Vector Kit; Stratagene, La Jolla, CA, USA) or the pCC1FOS fosmid vector (CopyControl Fosmid Library Production Kit; Epicentre, Madison, USA). Plasmid DNAs were isolated using two BioRobots8000 (QIAGEN GmbH, Hilden, Germany). All plasmids were end-sequenced using the following primers: Forward and Reverse on ABI sequencer ABI3730XL or ABI377 (Applied Biosystems, Foster City, CA, USA), NewForward and NewReverse on MegaBace sequencer MB4000 (GE Healthcare, Munich, Germany), cos_for and cos_rev for cosmid vectors, and pCC1_Forward and pCC1_Reverse for fosmid vectors (costumn primers see Table S5). Sequences were produced using either ABI BigDye Terminator 3.1 chemistry (Applied Biosystems) or ET-Terminator chemistry (GE Healthcare). About 104 000 generated sequences were assembled into contigs using the Phrap assembly tool (http://www.phrap.org). Primer walking on plasmids, cosmid clones and fosmid clones as well as PCR-based techniques were used to close remaining gaps and to solve misassembled regions caused by the high number of repetitive sequences. Mainly the sequences derived from the over 2000 fosmid clones served for verification of orientation, linkage and consistency of all contigs. All manual editing steps were performed using the STADEN software packages and the Gap4 versions therein (Staden, 1996; Staden *et al*., 2000). After sequence polishing and finishing, the genome sequence had a 11.8-fold sequence redundancy. The final chromosome sequence was assembled from 110 511 reads and the plasmid from 3572 reads with a minimum PHRED score of 15 and an average used read length of 615 bp.

### Prediction and annotation of CDS, gene family identification

Prediction of open reading frames (ORFs) or coding sequences (CDS) was accomplished with YACOP (Tech and Merkl, 2003) using the ORF-finding programs Glimmer (Delcher *et al*., 1999a,b), Critica (Badger and Olsen, 1999) and *Z* curve (Guo *et al*., 2003). Prediction of coding sequences within GenDB 2.0 to 2.4 (Meyer *et al*., 2003; CeBiTec, University of Bielefeld, Germany) was accomplished using the ORF-finding programs Critica (Badger and Olsen, 1999), Gismo (Krause *et al*., 2007), Glimmer (Delcher *et al*., 1999a,b) and reganor (McHardy *et al*., 2004). The ERGO software package (Overbeek *et al*., 2003) licensed by Integrated Genomics (Chicago, IL, USA) was used for manual curations of all CDS by comparing the predicted protein sequences with the publicly available data sets of SWISSPROT, GenBank, ProDom, COG and Prosite (Falquet *et al*., 2002). The CD-Search software (Marchler-Bauer and Bryant, 2004) was used to screen all CDS for similarities to known protein families and domains. The TMpred software was used for the prediction of transmembrane helices within the CDS (Hofmann and Stoffel, 1993). Prediction of regulatory proteins was performed by screening the whole genome protein database of *Db. autotrophicum* HRM2 with appropriate HMM models. The models for prediction of histidine kinases have been extracted from Pfam (Bateman *et al*., 2004; Galperin *et al*., 2001; Finn *et al*., 2006). Appropriate models of ECF σ-factors have been developed on the basis of a compilation of all known ECF σ-factors (Staron *et al.*, 2007). Putative genomic islands, referred to as alien genes and/or highly expressed CDS, were searched with a score-based FSIGI-HMM program (Merkl, 2004; Waack *et al*., 2006).

### Comparative genomics and repeat analysis

Repeated elements within the genomic sequence were calculated and analysed using the programs repfind and repvis, which both are part of the reputer software package (Kurtz *et al*., 2001). Pairwise graphical alignments of whole genome assemblies (e.g. synteny plots) were generated using the MUMmer system (Delcher *et al*., 1999a,b). The protein sequences encoded by *Db. autotrophicum* HRM2 were used for bidirectional blast comparisons among a selected representative set of 700 whole genome protein data sets, thus identifying putative orthologous genes. Bidirectional blast for bacterial genomes (BiBaG) has been performed using a variant method of blast developed by Antje Wollherr and Heiko Liesegang (pers. comm.). The similarities of putative orthologous proteins from 67 taxonomically diverse genomes were displayed by a colour code (see also Fig. 4). Paralogous genes have been determined by a ‘all against all’blast of all proteins within a genome. A pair of genes has been considered as paralogous if the determined protein sequences share at least 50% sequence identity in an alignment of 80% length of the shorter sequence (Table 1). Detailed results of all blast comparisons are available in the Table S1.

## Supporting information

**Fig. S1.** Genome circle. Circles (from outside to inside): 1, the outer circle gives the location of all genes of the C1 pathway (red), the heterodisulfide reductase activities (blue) and the methylviologen non-reducing hydrogenase activities (green). Arrows above indicate the orientation of the marked genes with respect to the deduced origin of replication; 2, scale circle in Mbp; 3, the third ring gives the location of all ORFs on the leading and the lagging strand, classified and colour-stained as given by the Clusters of Orthologous Groups of proteins (COG) (http://www.ncbi.nlm.nih.gov/COG/grace/fiew.cgi); 4, all genes that differ significantly from the standard codon usage of *Db. autotrophicum* HRM2 and therefore represent putative foreign genes are marked in green; 5, on the fifth ring all transposase/putative transposonrelated genes (dark blue), or recombinase/invertase genes (orange) are marked; arrows above indicate the orientation of the given genes with respect to the origin of replication; 6, the GC content (red) is given with marked average value (black); 7, translational components are given: rDNA clusters (green), incomplete rDNAs (red) and all tRNAs (blue); arrows above indicate the orientation of the marked genes with respect to the origin of replication.

**Fig. S2.** Mummer plot. Comparison of the *Db. autotrophicum* HRM2 chromosome with the chromosomes of the closely related δ-proteobacteria *Dt. psychrophila* LSv54, *Dv. vulgaris* Hildenborough, *Dv. desulfuricans* G20, *G. sulfurreducens* PCA and the sulfate-reducing archaeon *A. fulgidus* VC-16. Red dots indicate colinear similarities, green dots indicate inverted similarities. The chromosome of *Db. autotrophicum* HRM2 completely lacks synteny with the compared chromosomes. In contrast, a mummer plot of a comparison of *Dv. vulgaris* Hildenborough and *Dv. desulfuricans* G20 reveals genome synteny. All comparisons were performed with the programs from the MUMmer software package as described in the documentation for distantly related sequences (http://mummer.sourceforge.net/).

**Fig. S3.** Protein domain alignment of heterodisulfide reductases (Hdrs) from *Db. autotrophicum* HRM2 and experimentally characterized Hdr reference proteins. A multiple protein domain alignment of all Hdrs from Db. autotrophicum HRM2 and experimentally characterized reference proteins revealed the four distinct Hdr groups A, L, D and F. The alignment is based on colour-coded Pfam domain scans. Each protein group contains at least one selenocysteine-containing and one non-selenocysteine-containing paralogue of the enzyme. In many cases *Db. autotrophicum* HRM2 encodes the protein domains necessary to form an Hdr by two or more colocalized genes. It is remarkable that in each case the order of the Hdr domains is conserved in the order of the encoding genes and is colinear to the order of domains within the reference proteins. Group A contains four Hdrs of *Db. autotrophicum* HRM2 with similarities to HdrA of the archaea *M. mazei* and *A. fulgidus*. HdrA1 to HdrA3 are encoded by two and three genes, respectively, which contain all Pfam domains found in the single polypeptides of the reference proteins. For HdrA4 no protein encoding the MvhD domain could be identified. Group L contains three members, HdrL1 to HdrL3, each colocalized with a MvhD protein. The group L proteins are newly found in *Db. autotrophicum* HRM2. Group D contains HdrD1 to HdrD5 from *Db. autotrophicum* HRM2. The members of this group align with the domains of HdrD1 from *A. fulgidus*. HdrD is a paralogue of HdrD from HdrDE from *M. mazei* and *A. fulgidus*, which are encoded by one protein in the case of *A. fulgidus* and two proteins in the case of *M. mazei* respectively. An orthologue of HdrE could not be identified within the *Db. autotrophicum* HRM2 genome. Group F consists of three members HdrF1 to HdrF3. HdrF1/F1′ is encoded by two genes whereas HdrF2 and HdrF3 are encoded by one gene. Group F represents, as group L, a newly found type of Hdrs from *Db. autotrophicum* HRM2.

**Table S1.** Bidirectional blast comparisons of 4947 protein sequences from *Db. autotrophicum* HRM2 and 700 whole genome protein data sets.

**Table S2.** Repeat analysis of sulfate- or sulfur-reducing prokaryotes.

**Table S3.** Selenocysteine-containing proteins.

**Table S4.** Pfam domain occurrence in two-component systems from *Db. autotrophicum* HRM2.

**Table S5.** Sequence of used primers.
