# Supplementary figures and images for: Genome sequence of Desulfobacterium autotrophicum HRM2, a marine sulfate reducer oxidizing organic carbon completely to carbon dioxide
Source: Environ Microbiol. 2009 May;11(5):1038–55. doi: 10.1111/j.1462-2920.2008.01825.x (PMC2702500; doi:10.1111/j.1462-2920.2008.01825.x)

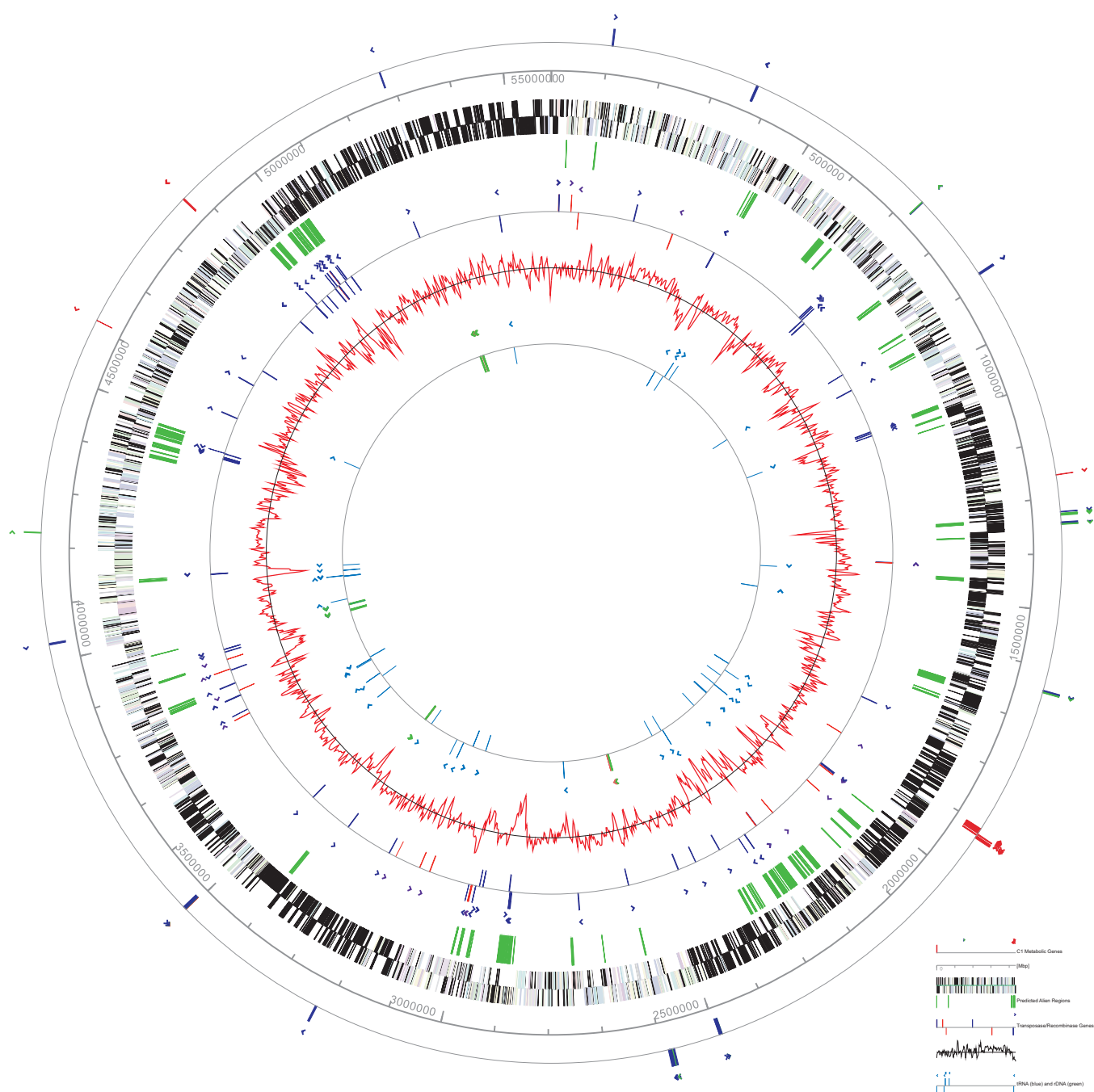

Figure S1

Supplement: Supplementary file 1 [file emi0011-1038-SD1.pdf]

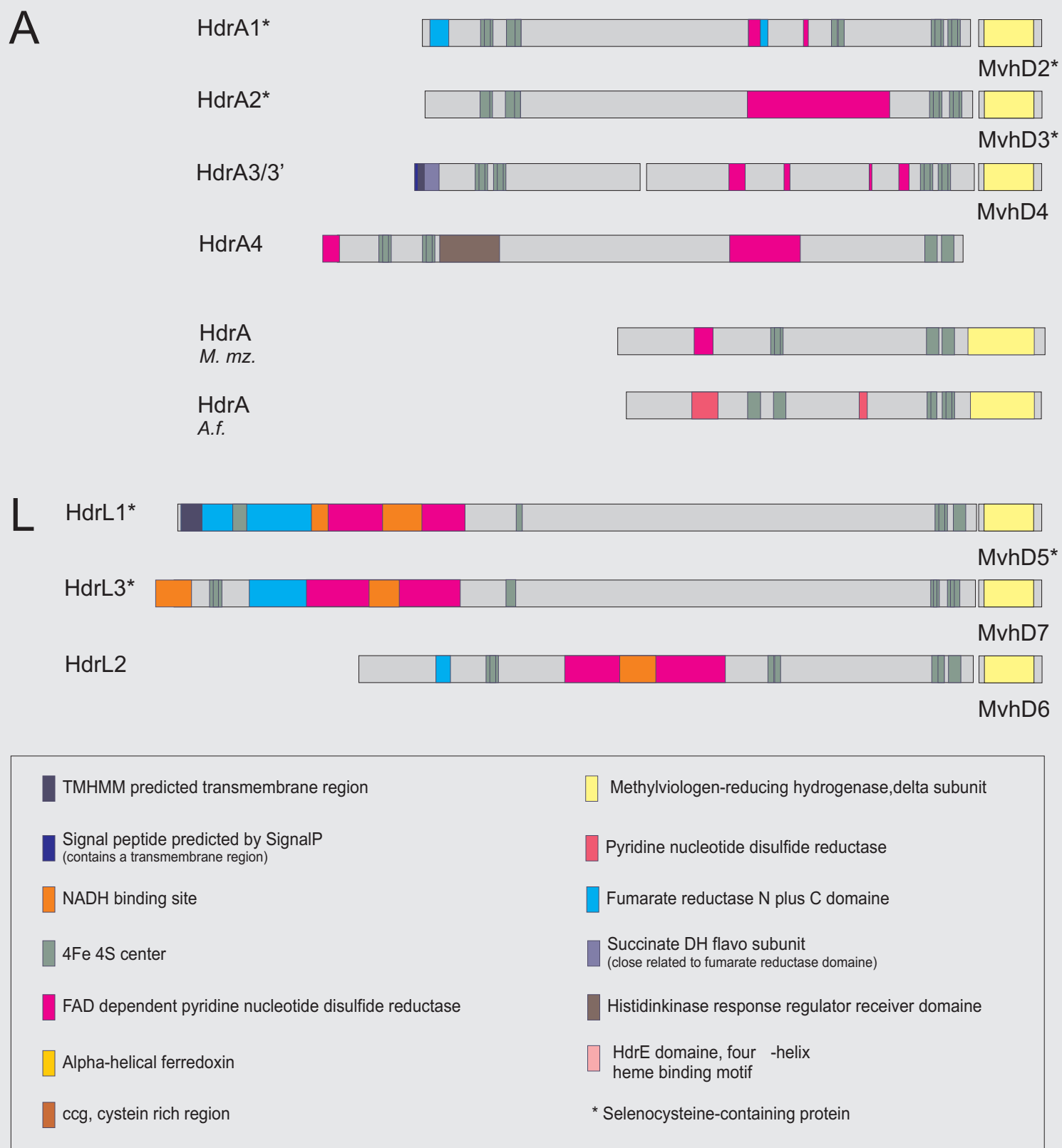

Fig. S3a

Supplement: Supplementary file 3 [file emi0011-1038-SD3a.pdf]

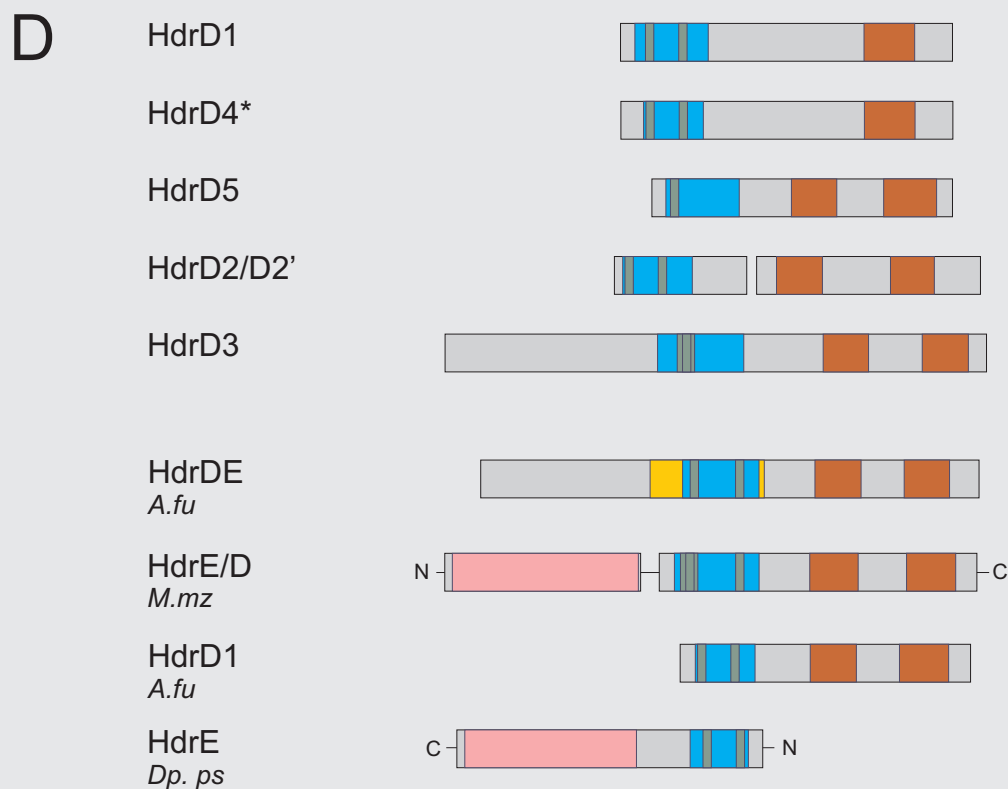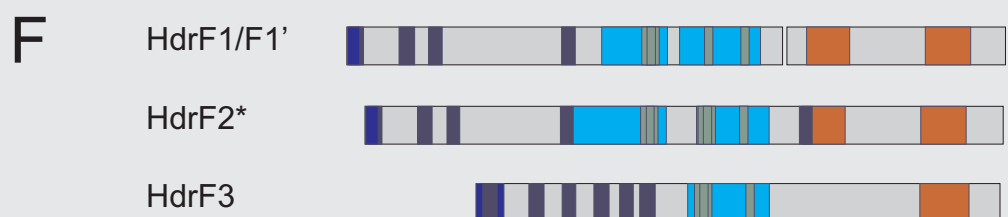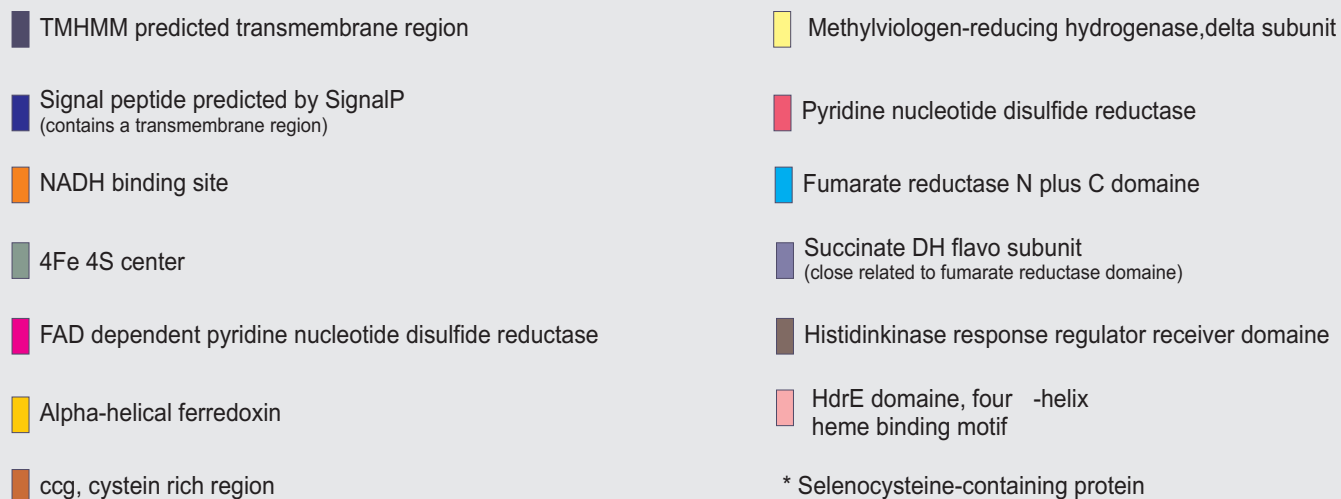

Fig. S3b

Supplement: Supplementary file 4 [file emi0011-1038-SD3b.pdf]
