# Supplementary material for: Genome sequence of Desulfobacterium autotrophicum HRM2, a marine sulfate reducer oxidizing organic carbon completely to carbon dioxide
Source: Environ Microbiol. 2009 May;11(5):1038–55. doi: 10.1111/j.1462-2920.2008.01825.x (PMC2702500; doi:10.1111/j.1462-2920.2008.01825.x)
Supplement: Supplementary file 2 [file emi0011-1038-SD2.pdf]

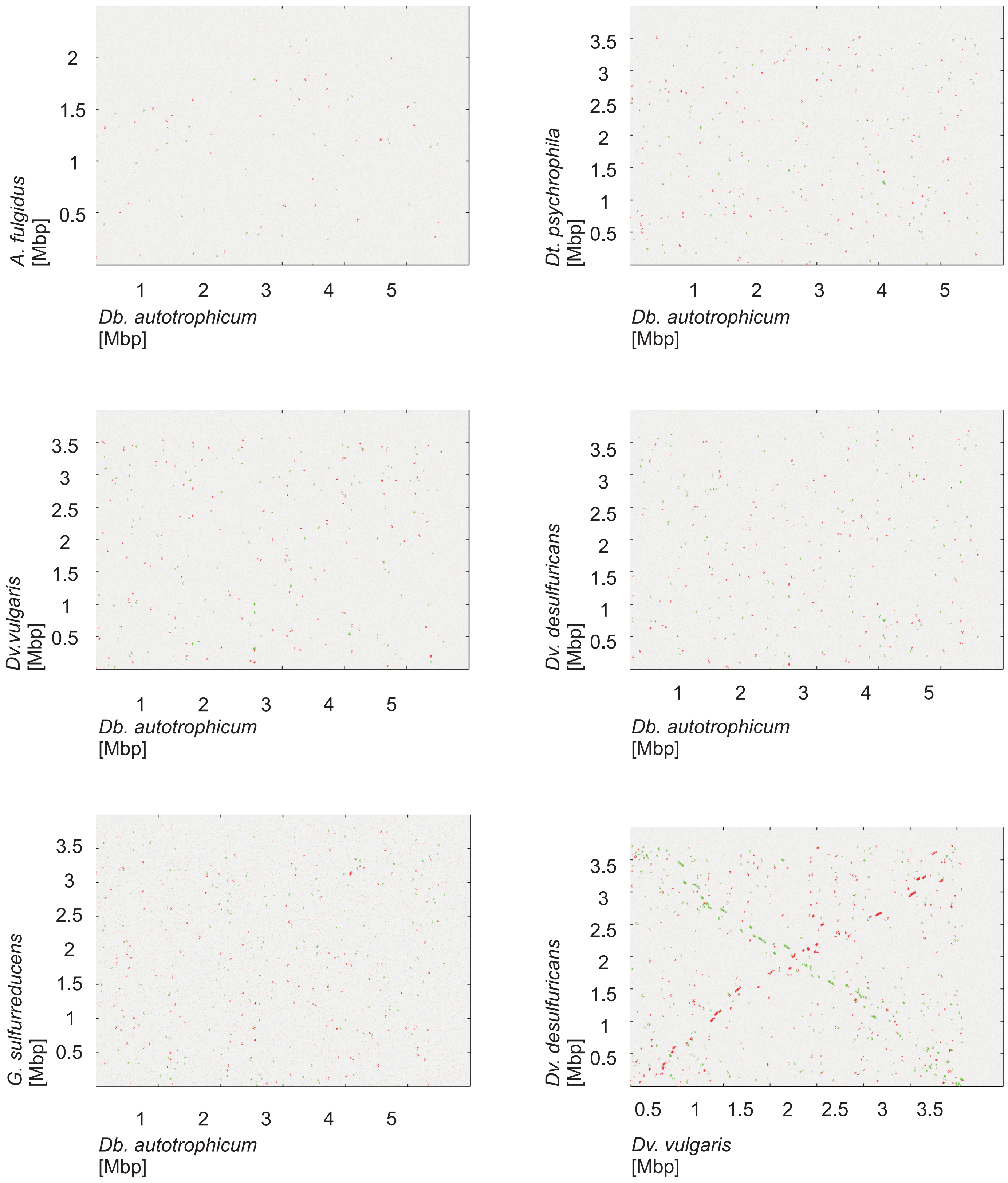

Figure S2 – Mummer Plot:

Comparison of the *Db. autotrophicum* chromosome with the chromosomes of the closely related δ-proteobacteria *Dt. psychrophila*, *Dv. vulgaris*, *Dv. desulfuricans*, *G. sulfureducens* and the sulfate reducing archeon *A. fulgidus*. Red dots indicate co-linear similarities, green dots indicate inverted similarities. The chromosome of *Db. autotrophicum* completely lacks synteny with the compared chromosomes. In contrast, a mummer plot of a comparison of *Dv. vulgaris* and *Dv. desulfuricans* has been added which shows genome synteny. All comparisons were performed with the programs from the MUMmersoftware package as described in the documentation for distant related sequences (http://mummer.sourceforge.net/).
